# Neural correlates of emotion processing comparing antidepressants and exogenous oxytocin in postpartum depressed women: An exploratory study

**DOI:** 10.1371/journal.pone.0217764

**Published:** 2019-05-31

**Authors:** Tierney K. Lorenz, Hu Cheng, Julia R. Heiman

**Affiliations:** 1 Department of Psychology, University of Nebraska-Lincoln, Lincoln, Nebraska, United States of America; 2 Center for Brain, Biology and Behavior, University of Nebraska-Lincoln, Lincoln, Nebraska, United States of America; 3 Department of Psychological and Brain Sciences, Indiana University Bloomington, Bloomington, Indiana, United States of America; Radboud University Medical Centre, NETHERLANDS

## Abstract

Despite common use of antidepressants to treat postpartum depression, little is known about the impact of antidepressant use on postpartum brain activity. Additionally, although oxytocin has been investigated as a potential treatment for postpartum depression, the interaction between antidepressants and exogenous oxytocin on brain activity is unknown. We explored postpartum depressed women’s neural activation in areas identified as important to emotion and reward processing and potentially, antidepressant response: the amygdala, nucleus accumbens and ventral tegmental area. We conducted a secondary analysis of a functional imaging study of response to sexual, crying infant and smiling infant images in 23 postpartum depressed women with infants under six months (11 women taking antidepressants, 12 unmedicated). Participants were randomized to receive a single dose of oxytocin or placebo nasal spray. There was significantly higher amygdala activation to sexual stimuli than either neutral or infant-related stimuli among women taking antidepressants or receiving oxytocin nasal spray. Among unmedicated women receiving placebo, amygdala activation was similar across stimuli types. There were no significant effects of antidepressants nor oxytocin nasal spray on reward area processing (i.e., in the nucleus accumbens or ventral tegmental area). Among postpartum women who remain depressed, there may be significant interactions between the effects of antidepressant use and exogenous oxytocin on neural activity associated with processing emotional information. Observed effect sizes were moderate to large, strongly suggesting the need for further replication with a larger sample.

## Introduction

Antidepressants are a standard treatment for postpartum depression (PPD) [1]. However, the effect of antidepressants on the postpartum brain are understudied, as most studies of antidepressant action have investigated males only [2]. This is a major knowledge gap, given the sex/gender differences noted in antidepressant response [3] as well as in the systems that underlie the putative antidepressant mechanisms such as serotonin transport [4] and functional connectivity [5]. The experience of pregnancy, parturition, and providing maternal care may alter neuroendocrine function in ways that interact with antidepressant actions [6, 7].

Also, there is increasing interest in the impact of antidepressants on neuroendocrine systems relevant to PPD. In particular, oxytocin–a neuropeptide that mediates social behaviors such as maternal [8] and sexual behaviors [9]–may play a role in depression [10], particularly in postpartum [11]. In postpartum women, oxytocin appears to facilitate adaptive reorganization of key neural structures in the hypothalamus, hippocampus, and amygdala [12, 13]. Endogenous oxytocin during the postpartum period may also buffer against the negative effects of cortisol and other aspects of stress reactivity [14–16]–a much needed adaptation to a very stressful time of life. In fact, low endogenous oxytocin has been associated with risk of PPD [11, 17, 18].

As oxytocin may play a role in PPD, exogenous oxytocin administration has been proposed both as a primary treatment [19], or as an adjunctive to antidepressant treatment [20]. While rodent models suggest exogenous oxytocin may improve PPD-like symptoms [21], clinical trials in human mothers have not shown clear benefits [22–25]. These conflicting reports have generally either excluded women taking antidepressants or considered medicated and unmedicated women together, complicating interpretation. Moreover, the effects of oxytocin on antidepressant action in depressed mothers are potentially different from a general depressed population [26, 27], underscoring the need to examine interactions of oxytocin and antidepressant use in the context of PPD specifically.

As a secondary analysis of a previously collected dataset, we explored brain activity in women with PPD who were or were not taking antidepressants, and who received either placebo or an oxytocin nasal spray. While the sample size is small, exploring these data could reveal some clues for further study. Brain response data are scant for postpartum depressed women, and there is even less known about PPD women on antidepressants. Because of the vast amount of information collected during functional neuroimaging, it is particularly important to have as much specificity as possible in pre-defining analyses; this specificity relies on evidence from prior research. Thus, although the exploratory results in the present study are in and of themselves only suggestive, they could be critical to future research. As such, we explored neural activity in areas of most interest to researchers in the areas of emotion processing and antidepressant treatment mechanisms.

Prior meta-analyses of mixed groups of depressed men and women (non-postpartum) have indicated that antidepressant treatment is associated with changes in activation to visual emotional stimuli in the limbic system, including the amygdala, and increased activation in the mesolimbic “reward systems” including the nucleus accumbens (NAc) and ventral tegmental area (VTA) [28]. Antidepressant response in non-postpartum depression has been predicted by changes in activation to these areas [29–33]. The amygdala, VTA and NAc also appear to be sites of significant structural and functional change during the postpartum period [34, 35]. Not surprisingly, these areas become particularly neuroplastic in response to the increased oxytocin signaling during pregnancy and postpartum [36].

Although increased neuroplasticity is beneficial for adapting the brain to the new demands of motherhood, it may also contribute to increased risk of PPD if reorganization is disrupted (e.g., by significant life stressors [37, 38]), leading to persistently maladaptive patterns of activation [39]. This is particularly true for women with histories of early life stressors, as the degree to which oxytocin release leads to increased neuroplasticity may depend on prior stress experiences [40]. That is, women with a history of childhood trauma may be at increased risk of reproductive mood disorders associated with the increased neuroplasticity induced by oxytocin [41]. There is some evidence that antidepressants can reverse the structural modifications in the amygdala, VTA and NAc that may underlie PPD [37]. In rodent models of PPD, postpartum depressive-like behavior is associated with structural changes to the NAc and amygdala, but citalopram administration reverses these changes [42]. Similarly, while gestational stress appears to disrupt typical neuroplasticity in postpartum rats, these effects are reversed with fluoxetine treatment [37, 43]. Based on these data, we selected the amygdala, VTA, and NAc as our regions of interest (ROI): each has been shown relevant for antidepressant response (including in PPD), each undergoes significant re-organization during the postpartum period, and each has been shown to be responsive to oxytocin.

We hypothesized that, relative to unmedicated PPD women, PPD women taking antidepressants would exhibit lower amygdala activation to sexual stimuli. In a non-postpartum context, antidepressant use is associated with lower sexual interest in women [44], as well as lower amygdala response to sexual images [45]. Healthy (non-depressed) postpartum women have significantly lower amygdala response to sexual images than nulliparous women [46]; insofar as antidepressants result in bringing PPD women’s neural responses closer to those of healthy postpartum women, we should expect antidepressants to be associated with lower amygdala response to sexual images. Furthermore, *contrasting* amygdala responses to sexual vs. infant related stimuli may be a marker of initial neuroadaptation to motherhood, as increasing response to infant stimuli and decreasing response to sexual stimuli in the early months postpartum may reflect increased investment in the current offspring vs. potential new offspring [46, 47]. Thus, we additionally hypothesized that, for activation of the amygdala and reward areas (VTA, NAc), the *relative difference* between infant and sexual images would be greater in PPD women taking antidepressants than in unmedicated PPD women, in whom responses would be more muted.

As antidepressants have been shown to amplify reward activation in non-postpartum contexts [32, 48], we hypothesized that PPD women taking antidepressants would have significantly higher VTA and NAc activation to positive emotional images (smiling infants, sexual images) relative to unmedicated women. This means we expected different effects of antidepressants on amygdala vs. reward area processing of sexual images: decreasing activation in the former while increasing the latter. Such effects would parallel report of women’s subjective experience of postpartum sexuality: while women’s sexual interest decreases in the postpartum period, the degree of pleasure from sexual activity remains stable [49, 50].

Animal models suggest that oxytocin may mediate some of the antidepressant effects of SSRIs [51, 52], and data from clinical studies in humans show resting levels of oxytocin increase following antidepressant administration [53].These findings hint at possible parallel mechanisms underlying both antidepressant use and oxytocin response. Thus, we hypothesized that the effects of oxytocin on neural activation to emotional stimuli would differ in women who were taking vs. not taking antidepressants. If there are similar systems underlying the response to both antidepressants and oxytocin in postpartum depressed women, we should expect the effects of exogenous oxytocin to be relatively less noticeable among women taking antidepressants. However, given the dearth of prior research, we did not have a priori hypotheses regarding how the combination of exogenous oxytocin and antidepressants would impact contrasts between stimuli types, or differences in activation of amygdala vs. reward processing areas.

## Methods

The present study was a secondary analysis from a larger investigation of differences in neural activity between postpartum and nulliparous women; detailed description of study procedures can be found in the primary publications [46, 47, 54].

### Participants

Women who were 3–6 months postpartum, breastfeeding at >75% of feedings, were recruited and screened for depression with the Edinburgh Postnatal Depression Scale (EPDS, [55]). Women with a history of psychosis or manic episodes were excluded. A total of 23 currently depressed postpartum women (scoring ≥ 12 on the EPDS) completed a scanning session and provided complete data regarding medication use. Of these, 11 reported current use of an SSRI antidepressant (sertraline, *N* = 8, fluoxetine, *N* = 2, citalopram, *N* = 1), while 12 reported no antidepressant use. Both groups were similar in demographics (Table 1). Also, on the day of the experimental session, participants completed the Center for Epidemiologic Study–Depression scale (CES-D [56]); the medicated vs. unmedicated groups were similar in level of self-reported depressive symptoms at the time of scanning.

**Table 1:** Demographics. There were no significant differences in demographics across medication group. CESD: Center for Epidemiologic Study–Depression scale.

	Antidepressant group (n = 11)		Unmedicated group (n = 12)	
	*Mean*	*SD*	*Mean*	*SD*
**Age of participant (years)**	30.82	*4*.*94*	30.17	*5*.*47*
**CES-D Scale score on day of testing**	23.73	*9*.*00*	24.91	*12*.*13*
**Pre-trial urinary oxytocin (pg/mL)**	10.01	*6*.*64*	9.76	*6*.*74*
	*n*	*%*	*n*	*%*
**History of Psychotherapy**				
** No history**	2	*18%*	7	*58%*
** Past, not current**	2	*18%*	2	*17%*
** Current**	7	*64%*	3	*25%*
**Menstrual period returned?**				
** Yes**	8	*72%*	7	*58%*
** No**	3	*27%*	5	*42%*
**Race/Ethnicity**				
** White**	10	*91%*	8	*66%*
** Non-white**	1	*9%*	4	*33%*
**Highest Education**				
** High school**	3	*27%*	4	*33%*
** College degree**	5	*45%*	7	*58%*
** Postgraduate degree**	3	*27%*	1	*8%*
**Self-reported physical health**				
** Fair**	1	*9%*	2	*17%*
** Good**	9	*81%*	8	*66%*
** Excellent**	1	*9%*	2	*17%*
**Lifetime number of live births**				
** 1**	9	*82%*	8	*66%*
** 2**	2	*18%*	2	*17%*
** 3 or more**	0	*0%*	2	*17%*
**Other medications on day of testing**				
** None**	8		10	
** Hormonal contraceptives**	2		0	
** Antihistamines**	1		1	
** Lansoprazole**	0		1	
** Ibuprofen**	1		0	

### Experimental procedure

To reduce variability in endogenous oxytocin, participants nursed their infant ~1 hour 15 minutes prior to imaging. All participants were administered nasal spray approximately 30 min prior to imaging; nasal spray condition (oxytocin vs. placebo) was randomized and double-blinded. Oxytocin nasal spray contained an inert carrier solution and 24IU of synthetic oxytocin (Syntocinon, Novartis Pharma, Switzerland), while the placebo spray contained inert carrier only; these sprays have been shown to be indistinguishable to participants [57]. While the elimination half-life of peripheral (plasma) oxytocin is relatively short (~20 minutes [58]), concentrations in cerebrospinal fluid peak approximately 45–75 minutes of nasal spray administration [59].

Participants provided a urine sample before and after nasal spray application, which was tested for oxytocin. These tests confirmed a significant increase in urinary oxytocin in the oxytocin nasal spray group (*M*_*pre*_ = 7.29 pg/mL, *SD* = 2.14; *M*_*post*_ = 44.37 pg/mL *SD* = 46.27; paired *t*(10) = -2.63, *p* = .025), but not the placebo (*M*_*pre*_ = 12.34pg/mL, *SD* = 8.66; *M*_*post*_ = 11.34 pg/mL, *SD* = 11.80; paired *t*(10) = .25, *p* = .809). Baseline urinary oxytocin levels were similar across medication groups (antidepressant *M* = 10.30 pg/mL, *SD* = 6.86; unmedicated *M* = 9.76 pg/mL, *SD* = 6.74), and were in the same range reported in prior research on urinary oxytocin in postpartum women [60, 61]. Antidepressant use did not predict changes in urinary oxytocin (*F*(1, 24) = 1.14, *p* = .296).

Following nursing and nasal spray administration, participants viewed emotionally relevant visual stimuli in a functional magnetic resonance imaging (fMRI) paradigm (see below for imaging details). We examined blood oxygenation-level dependent (BOLD) responses to 4 stimuli types: sexually explicit images, smiling and crying infant images, and emotionally neutral images. The neutral images were derived from a set of International Affective Picture Set (IAPS) images validated to evoke low emotional arousal and fall in the middle of the valence range [47, 62]. Infant images were taken from publicly available websites and were validated to evoke moderate but significant emotional arousal; given a Likert scale of 1 (least intense) to 9 (most intense), the average rating for infant images was 4.70 [46]. Sexual images included images of nude heterosexual couples engaging in sexual acts (e.g., oral sex, vaginal intercourse) that were derived from a set of images previously shown to evoke sexual interest in women [46, 63]; these images similarly were rated as moderately but significantly emotionally arousing, with an average intensity rating of 4.35 [46]. During stimuli presentation, participants completed a backwards-matching task to ensure adequate attention. The study was approved by the Institutional Review Board at Indiana University Bloomington, and all participants provided written informed consent.

### Imaging procedures and data processing

The imaging session consisted of a 3 plane-localizing scan to determine slice volumes (10 sec), seven whole brain blood oxygenation-level dependent (BOLD) scans (5 min each), and a whole brain high-resolution anatomical scan (5 min), for a total of approximately 1 hour. Functional (BOLD) scans were started with a 12-sec at-rest baseline, followed by 64 randomized stimuli presented for 2 seconds each (with variable inter-stimuli intervals of 2–6 seconds).

Imaging was conducted in a Seimens Magnetom Trio 3T whole body MRI. All images were collected on a 32-channel phased-array head coil. The field of view of 220 × 220 mm. An in-plane resolution of 128 × 128 pixels, and 35 axial slices of 3.4 mm thickness per volume, produced voxels that were 1.7 × 1.7 × 3.4 mm. A gradient echo BOLD echo-planar imaging (EPI) sequence was used for capturing functional images, including the following parameters: TE = 24 ms, TR = 2,000 ms, flip angle = 70°. We used parallel imaging with an iPAT factor of 2. For anatomical volumes, we used high-resolution T1-weighted images, acquired with a Turbo-flash 3-D sequence, including the following parameters: TI = 900 ms, TE = 2.67 ms, TR = 1800 ms, flip angle = 9°, with 192 sagittal slices of 1 mm thickness, a field of view of 224 × 256 mm, and an isometric voxel size of 1 mm^3^.

We used BrainVoyager QX 2.2 to prepare imaging data for statistical analysis. Each participants’ anatomical volumes were stereotaxically transformed using the Talaraich atlas with an eight-parameter affine transformation. Using an intensity-based motion correction algorithm, functional volumes were realigned to the volume closest in time to the anatomic volume. We also corrected functional volumes using slice scan-time correction, 3-D spatial Gaussian filtering (FWHM 6 mm), and linear trend removal. These corrected functional volumes were co-registered to the relevant anatomical volume using an intensity-based matching algorithm, and normalized to the common stereotactic space with an eight-parameter affine transformation. Functional data were re-sampled to 3 mm^3^ isometric voxels. Beta weights of a random-effects general linear model (based on timing protocol of the blocked stimulus presentation, convolved with a two-gamma hemodynamic response function) were extracted from group ROIs using the VOI/ROI ANCOVA data table tool in BrainVoyager’s volume of interest module.

## Results

We conducted repeated measures ANCOVAs with stimulus type (neutral, sexual, infant crying, or infant smiling) as the repeated measures variable, medication use and nasal spray group (and their interaction) as fixed effects, and the following covariates: scores on the Center for Epidemiological Studies-Depression (CES-D) scale on the day of the imaging session [56], age, pre-trial urinary oxytocin level, and activation to nonsense images in which the pixels from the other stimuli were scrambled. Controlling for activation to nonsense stimuli accounted for individual differences in general activation to visual stimuli not related to emotion or reward processing. None of the covariates differed significantly between groups, but had considerable variance across the entire sample and were thus used to control for individual differences at baseline. We conducted separate models predicting activation in the left amygdala (Talairach coordinates: -19, -6, -10), right amygdala (Talairach coordinates: 15, -5, -9), left NAc (Talairach coordinates: 11, 12, -8); right NAc (Talairach coordinates: -13, 10, 8), and VTA (Talairach coordinates: 2, -23, -5).

### Amygdala

**Fig 1:**
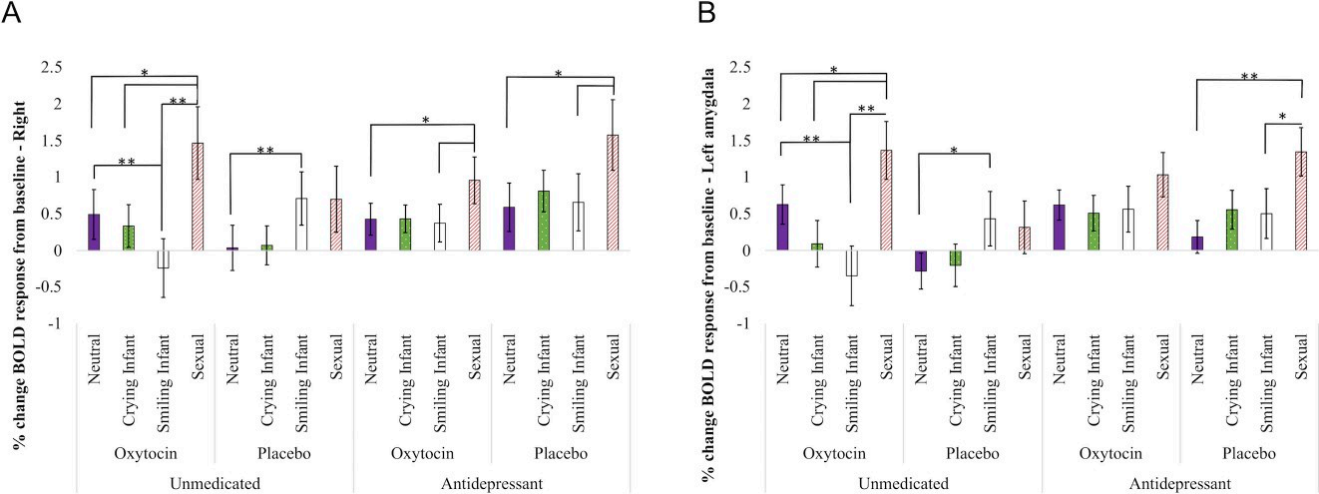
**a-b. Activation of amygdala (1a: Left; 1b: Right) to visual emotional stimuli, controlling for age, CES-D score, pre-trial urinary oxytocin, and activation to scrambled images**. Contrast bars represent significant differences between response to different stimuli types within the group (i.e., significant repeated measures effects). *: *p* < 0.05; **: *p* < 0.01.

The main effect of antidepressant use was non-significant (Left: *F*(1, 14) = 3.26, *p* = .09, η^*2*^_*partial*_ = 0.19; Right: *F*(1, 14) = 1.052, *p* = .32, η^*2*^_*partial*_ = 0.07) as was nasal spray condition (Left: *F*(1, 14) = 0.15, *p* = .71, η^*2*^_*partial*_ = 0.01; Right: *F*(1, 14) = 1.052, *p* = .32, η^*2*^_*partial*_ = 0.07). In other words, neither antidepressant use nor oxytocin administration were associated with *overall* higher or lower amygdala activation. However, there was a significant interaction between medication, nasal spray condition, and stimuli type (Left: *F*(2, 13) = 6.85, *p* = .01, η^*2*^_*partial*_ = 0.51; Right: *F*(2, 12) = 3.67, *p* = .04, η^*2*^_*partial*_ = 0.48; see Fig 1); thus, we conducted follow-up contrasts to examine the nature of the interaction (see below).

#### Post-hoc specific contrast tests of amygdala activation by stimuli type

See Table 2 for all specific contrasts; general patterns are summarized below.

**Table 2:** Post-hoc simple contrasts between stimuli types by antidepressant use and nasal spray group. Significant within-group contrasts are highlighted in grey.

Contrasts	Antidepressant x placebo nasal spray				Antidepressant x oxytocin nasal spray				Unmedicated x placebo nasal spray				Unmedicated x oxytocin nasal spray			
	*M*_*diff*_	*SE*	*p*	*d*	*M*_*diff*_	*SE*	*p*	*d*	*M*_*diff*_	*SE*	*p*	*d*	*M*_*diff*_	*SE*	*p*	*d*
***Right amygdala***																
Neutral vs. crying infant	-0.22	0.37	0.56	0.22	-0.01	0.24	0.98	0.02	-0.03	0.34	0.92	0.03	0.16	0.38	0.68	0.16
Neutral vs. smiling	-0.07	0.20	0.73	0.13	0.05	0.13	0.69	0.15	-0.67	0.18	<0.01	1.41	0.73	0.20	<0.01	1.38
Neutral vs. sexual	-0.99	0.37	0.02	1.01	-0.53	0.24	0.05	0.83	-0.66	0.34	0.07	0.73	-0.98	0.38	0.02	0.97
Crying infant vs. smiling infant	0.15	0.43	0.73	0.13	0.06	0.28	0.84	0.08	-0.64	0.40	0.13	0.60	0.57	0.44	0.21	0.49
Crying infant vs. sexual	-0.77	0.45	0.11	0.65	-0.53	0.30	0.10	0.67	-0.63	0.42	0.16	0.57	-1.13	0.47	0.03	0.91
Smiling infant vs. sexual	-0.92	0.40	0.04	0.87	-0.58	0.26	0.04	0.84	0.01	0.37	0.98	0.01	-1.71	0.41	<0.01	1.58
	***M***_***diff***_	***SE***	***p***	***d***	***M***_***diff***_	***SE***	***p***	***d***	***M***_***diff***_	***SE***	***p***	***d***	***M***_***diff***_	***SE***	***p***	***d***
***Left amygdala***																
Neutral vs. crying infant	-0.37	0.27	0.19	0.52	0.11	0.25	0.66	0.17	-0.08	0.29	0.79	0.10	0.54	0.32	0.12	0.64
Neutral vs. smiling	-0.32	0.23	0.20	0.53	0.06	0.22	0.80	0.10	-0.72	0.26	0.01	1.05	0.97	0.28	<0.01	1.31
Neutral vs. sexual	-1.16	0.26	<0.01	1.69	-0.41	0.24	0.11	0.65	-0.6	0.29	0.06	0.78	-0.74	0.32	0.03	0.87
Crying infant vs. smiling infant	0.05	0.33	0.88	0.06	-0.06	0.31	0.86	0.07	-0.64	0.36	0.10	0.67	0.44	0.40	0.29	0.42
Crying infant vs. sexual	-0.79	0.43	0.09	0.69	-0.53	0.39	0.20	0.51	-0.52	0.47	0.29	0.42	-1.28	0.51	0.03	0.95
Smiling infant vs. sexual	-0.85	0.31	0.02	1.04	-0.47	0.29	0.13	0.61	0.12	0.34	0.74	0.13	-1.71	0.38	<0.01	1.70

The contrast of infant vs. sexual stimuli was of particular interest as a possible marker of neuroadaptation to motherhood. The contrast between smiling infant vs. sexual stimuli was significant in all groups *except* unmedicated women receiving placebo. That is, for women taking antidepressants and/or receiving oxytocin nasal spray, there was significantly greater amygdala activation to sexual stimuli than smiling infant stimuli; however, for unmedicated women receiving placebo there was no significant difference between stimuli types. The contrast between crying infant and sexual stimuli was significant only among unmedicated women receiving oxytocin nasal spray; in this group amygdala activation to sexual stimuli was significantly greater than to crying infant stimuli.

Contrasts between neutral and smiling infant stimuli were significant in unmedicated women; however, the direction of this contrast differed by nasal spray group. In unmedicated women receiving placebo, amygdala activity to smiling infant stimuli was significantly higher than to neutral stimuli; however, among unmedicated women receiving oxytocin nasal spray, amygdala activation to smiling infant stimuli was significantly *lower* than to neutral stimuli.

Finally, contrasts between neutral and crying infant stimuli, and between crying and smiling infant stimuli, were non-significant across all groups.

### Nucleus accumbens

**Fig 2:**
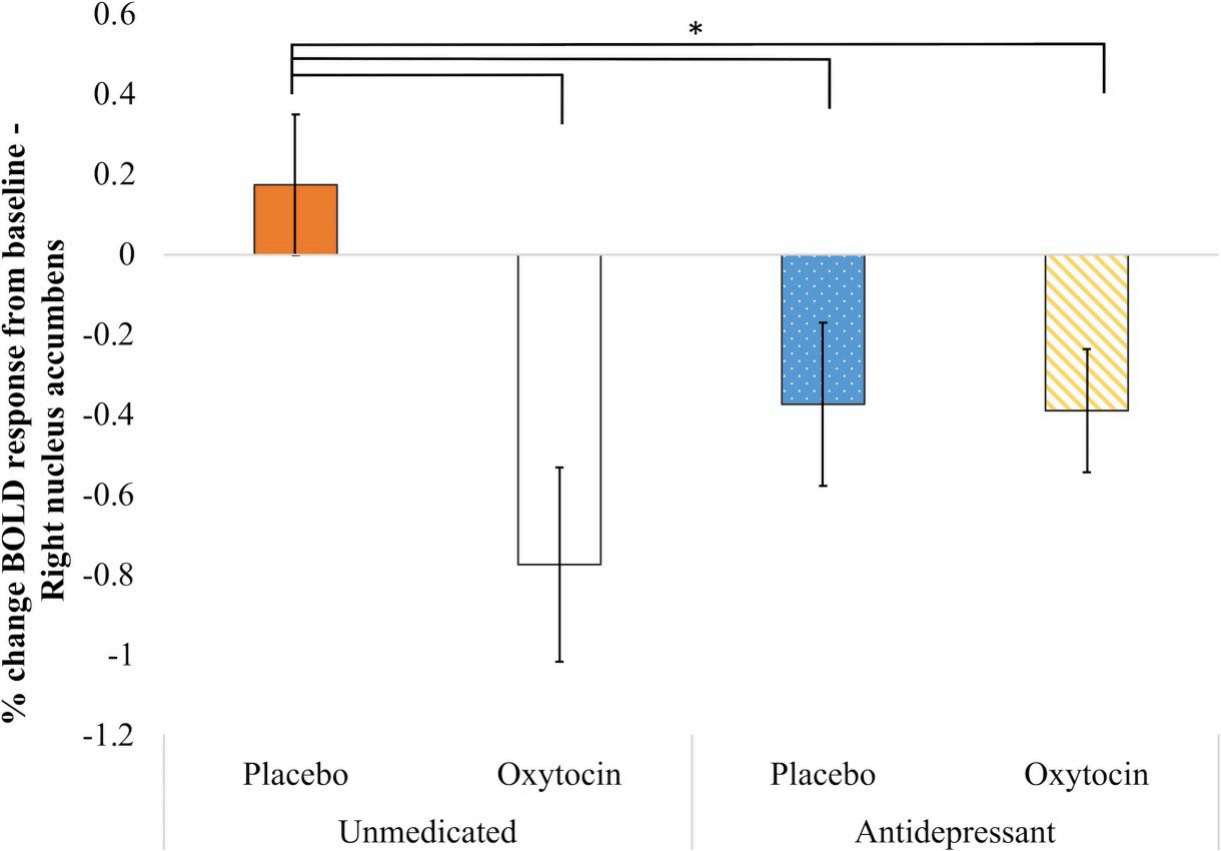
Activation of right nucleus accumbens to visual emotional stimuli, controlling for age, CES-D score, pre-trial urinary oxytocin, and activation to scrambled images. There were no significant contrasts between different stimuli types. *: *p* < 0.05.

The interaction between stimuli type, nasal spray condition and medication use on activation of the NAc was non-significant (Left: *F*(2, 14) = 1.91, *p* = .18, η^*2*^_*partial*_ = 0.32; Right: *F*(2, 14) = 0.36, *p* = .78, η^*2*^_*partial*_ = 0.08). However, there was a significant interaction between nasal spray condition and antidepressant use in activation of the right NAc (*F*(1, 14) = 5.66, *p* = .03, η^*2*^_*partial*_ = 0.29), such that unmedicated women receiving placebo nasal spray had significantly higher right NAc activation to all stimuli than women receiving antidepressants and/or oxytocin nasal spray (Fig 2).

### Ventral tegmental area

There were no significant effects by stimuli type, nasal spray condition, or medication use, nor any significant interaction in the VTA.

## Discussion

We explored if antidepressant use and exogenous oxytocin administration were associated with neural activation in postpartum depressed women. There were significant interactions between stimuli type, (non-randomized) antidepressant use, and (randomized) exogenous oxytocin administration in amygdala activation. However, antidepressant use and oxytocin administration were not associated with activation in reward processing areas, such the ventral tegmental area and the nucleus accumbens.

Only three studies have directly compared neural activity in depressed women who were vs. were not taking antidepressants. Briceño et al. [64] found no significant differences in overall neural response to facial emotional stimuli in depressed women who were and were not taking antidepressants. However, as that study did not predesignate ROIs, it was not designed to detect anything but very large whole-brain group differences. Yang et al. [65] examined changes in neural activation to sexual stimuli before and after antidepressant treatment in 7 depressed non-postpartum women. Following antidepressant treatment, participants significantly increased activation in the subcortical reward areas, notably the hypothalamus, septal nuclei and parahippocampal gyrus, but not the amygdala. However, there may be important differences in postpartum vs. non-postpartum women’s brain activity, particularly when considering reproductively relevant stimuli (such as the sexual stimuli used here). In non-postpartum women, antidepressants suppress sexual response [44], but as seen in Fig 1, in our sample of depressed *postpartum* women, women taking antidepressants did not have systematically lower amygdala response to sexual stimuli compared to unmedicated women.

Wonch et al. [66] examined amygdala response to infant stimuli in depressed and non-depressed postpartum mothers. Within their PPD group, there were no significant differences in amygdala response among 13 medicated vs. 18 unmedicated women. However, these authors did not include sexual stimuli, which may elicit stronger amygdala response [46]. Also, unlike in Wonch et al [66]’s paradigm, all the participants in our study nursed their infants prior to imaging; it is possible that recent exposure to *endogenous* oxytocin may moderate important differences between medication groups. Finally, the participants in our sample also reported significantly higher EDPS scores (*M* = 13.32, SEM = 0.79) than those studied by Wonch et al (*M* = 8.29, SEM = 0.84); possibly, the observed effects only emerge during more severe depressive episodes.

Importantly, there were significant interactions between oxytocin administration and antidepressant use in amygdala responsivity to emotional stimuli. Unmedicated women and women taking antidepressants responded differently to exogenous oxytocin administration, suggesting the need for caution as oxytocin is evaluated as an adjunctive to antidepressant treatment in PPD. There is evidence that depression is associated with lower responsivity to positively valenced emotional stimuli [67]. In healthy non-depressed women, administration of escitalopram is associated with lower amygdala activation to negative stimuli and higher activation to positive stimuli [68]. These patterns are thought to indicate that antidepressants attenuate hyper-reactivity to negative or stressful stimuli, while increasing reward salience associated with positive stimuli [69]. Oxytocin, however, appears to inhibit serotonin signaling in the dorsal raphe nucleus via suppression of activity in the amygdala [70]. As such, it is possible that instead of *amplifying* the effects of antidepressants on amygdala response to positive emotional stimuli (as would be beneficial in an adjunctive treatment), oxytocin may *attenuate* antidepressant response. As seen in Fig 1, we found that among PPD women taking antidepressants, oxytocin vs. placebo administration was associated with what appears to be *lower* amygdala activation to sexual stimuli, and no difference in smiling infant stimuli–at a minimum, we did not find evidence for increased amygdala responsiveness to positive emotional stimuli. However, when considering the possible treatment efficacy of adjunctive oxytocin there may be different effects of short-term and long-term exposure to exogenous oxytocin; the data from the present study can only speak to the short-term effects of a single administration. It is possible that longer-term joint oxytocin/antidepressant administration would have down-stream effects that could amplify therapeutic response, such as increasing production of neurotrophic factors [21] or epigenetic changes in the expression of genes for key receptors [71].

Unexpectedly, there were few significant associations of antidepressant use or oxytocin administration with reward area processing. We observed only one such association (namely, that unmedicated women receiving placebo had higher right NAc activity overall than any other group); as this association was not replicated bilaterally, it should be interpreted cautiously. There is evidence that the left NAc has greater involvement in reward-related activation than the right [72], which may lead to different thresholds for detecting depression-related attenuation of reward processing. For example, one study of unmedicated depressed individuals (50% female) found reduced activation in the left but not right NAc [73]. It is possible that the higher sensitivity of the left NAc may have influenced our findings in the left but not right NAc. It is also possible that due to our sample size, we missed some small but potentially relevant effects in the right NAc and VTA. Alternatively, it is possible that antidepressant and oxytocin doses, or the length of exposure, were insufficient to see significant associations in reward areas. Notably, all of the women in this study were depressed at the time of imaging, suggesting incomplete treatment response among the women taking antidepressants: we may have seen greater results if participants were on a higher dose that better managed their depressive symptoms.

The fact that we *did* see significant associations in the amygdala might suggest that the amygdala was relatively more responsive to the effects of antidepressants and/or oxytocin than the NAc or VTA. However, studies in animal models suggest the opposite: antidepressant administration is associated with significant changes in the organization and activation of the NAc but not the basolateral amygdala [42]. It is possible that the stimuli we used may have elicited an emotional, but not necessarily *rewarding*, response. Of the few studies that have found a significant effect of oxytocin administration on reward area processing in postpartum women, most have used stimuli of the participant’s own infants [74–76]–a much more salient and arguably more rewarding stimulus than stimuli of other people’s infants. It is also possible that when participants nursed shortly prior to imaging, the natural rise in prolactin levels associated with lactation may have temporary suppressed dopamine production which in turn may have limited activation of these dopaminergic pathways [77] (but see also [78]). Finally, it is possible these findings reflect a true null, in which neither oxytocin nor antidepressants are associated with significant effects in reward processing areas among PPD women. If so, this would require antidepressants to exert their therapeutic effect on depression via some other process–for example, by improving corticolimbic connectivity [79] or decreasing the effect of stress on functional reorganization during the postpartum period [42].

Our sample size was small; however, it is in keeping with similar studies [66], and we observed a number of significant associations with medium to large effect sizes. These effect sizes may serve as a reference point for future researchers in planning studies on neural activation in PPD women. They also signal the need for caution in interpreting null effects. In a few instances (e.g., the main effect of antidepressants on amygdala activation across stimuli), the observed associations were not *statistically* significant but the effect sizes were large enough to suggest a larger sample would achieve significance. Overall, we view the effect sizes observed in this analysis as large enough to suggest that further replication would be fruitful in revealing important group-wise differences. In particular, it will be important to collect samples large enough (and with enough precision) to examine the sub-regions of amygdala that contribute to the observed differences associated with antidepressant use. Such analyses can reveal important clues as to the mechanisms by which antidepressants exert their effects. For example, is a growing literature that suggests that, regardless of treatment modality, antidepressant effects are driven by neuroplastic changes specific to the basolateral amygdala [80–82]; however thus far this literature has not extended to PPD.

This was an observational study, and antidepressant use was not randomized; thus our conclusions must be tempered regarding the effects attributable solely to antidepressants. We cannot conclude antidepressants are causally related to *changes* in neural activity. Lacking randomization with a control group (or other means of controlling for the effect of treatment), it is difficult to conclude what might be driving the differences described here. However, it should be noted that randomizing PPD women to placebo (or no treatment) would raise ethical issues, and would only be warranted if there were sufficient evidence of the need for such a trial–again, highlighting the importance of preliminary findings such as those presented here. It is also possible that there were important differences between the participants who were and were not taking antidepressants. Both groups reported similar CES-D scores, suggesting that the women taking antidepressants may have had a more severe underlying depression that only partially remitted in response to medication. To address these limitations, future research would benefit from a design comparing a larger sample of PPD participants before and after successful antidepressant treatment.

Further research is also needed to examine if antidepressant use during the postpartum transition influences neuroadaptation: it has been proposed that increasing activation to infant-related stimuli and decreasing activation to sexual stimuli may reflect the shift of emotional processing resources from sexual interest to the demands of caring for an infant [47]. Our data suggested differences between women who were medicated vs. unmedicated in contrasts between reproductively relevant stimuli (i.e., sexual vs. infant-related stimuli), which supported the view that PPD represents a maladaptation to the unique challenges of motherhood. These findings, alongside a growing literature in evolutionary medicine [46, 47, 54, 83], underscore the need to consider the reproductive context in which PPD occurs when evaluating potential treatments.

## Conclusions

We compared neural activation in women with PPD who were and were not taking antidepressants. Among PPD women receiving oxytocin nasal spray and/or those taking antidepressants, there was significantly higher amygdala activation to sexual stimuli than to either neutral or smiling infant stimuli. However, among unmedicated PPD women receiving a placebo nasal spray, amygdala activation to sexual stimuli was not significantly different from activation to either neutral or infant-related stimuli. There was no consistent effect of antidepressant use or exogenous oxytocin administration in activation of reward areas (NAc, VTA). These data will help inform and encourage further attention to the growing body of research on the effects of psychoactive medication on neural function during the postpartum reproductive transition.

## Supporting information

S1 File BOLD response data. (SAV)
